# Mycotoxins in milk: Occurrence and evaluation of certain detoxification attempts

**DOI:** 10.1002/fsn3.3254

**Published:** 2023-04-03

**Authors:** Nesrine H. Youssef, Mohamed H. El Gammal, Hayman A. A. Altaie, Alaa Qadhi, Vincenzo Tufarelli, Caterina Losacco, Mohamed E. Abd El‐Hack, Nader R. Abdelsalam

**Affiliations:** ^1^ Regional Center for Food and Feed Dekhila Port Alexandria Egypt; ^2^ Agricultural Research Center Alexandria Egypt; ^3^ Regional Center for Food and Feed Agricultural Research Center Ghiza Egypt; ^4^ Department of Medical Laboratory Techniques, College of Medical Technology Al‐Kitab University Kirkuk Iraq; ^5^ Clinical Nutrition Department, Faculty of Applied Medical Sciences Umm Al‐Qura University Makkah Saudi Arabia; ^6^ Department of Precision and Regenerative Medicine and Jonian Area, Section of Veterinary Science and Animal Production University of Bari ‘Aldo Moro’ Valenzano Italy; ^7^ Poultry Department, Faculty of Agriculture Zagazig University Zagazig Egypt; ^8^ Agricultural Botany Department, Faculty of Agriculture (Saba Basha) Alexandria University Alexandria Egypt

**Keywords:** milk, mycotoxins, plant fibers bio‐binders, shacking, soaking

## Abstract

Milk contaminated with mycotoxins is a significant issue affecting human health, especially in infants. The current study aimed to investigate the presence of mycotoxins in milk collected from women farmers' vendors (WFV), and to evaluate certain herbal plant fibers as green mycotoxin binders. Moreover, explore the binding efficiency ratios of mycotoxins using shaking or soaking process incorporated with herbal extracts. Furthermore, compare the taste evaluations of tested milk are enriched with herbal extracts. Results indicated that the fumonisins were not detected in the collected cow milk samples but realized a 25% occurrence ratio in buffalo's milk samples. A high occurrence ratio of aflatoxin M1 (aflaM1) was observed in buffalo and cow milk samples. The soaking process of plant fibers in contaminated milk overnight significantly degrades and adsorbs mycotoxins particles. The shacking process incorporated with plant fibers exhibited more effectiveness in mycotoxins degradation than soaking or shacking processes alone. The speed of shacking process played an important role in the mycotoxin's binding process. All the tested plant fibers effectively reduced all mycotoxin presence in contaminated milk, especially green tea, during the soaking or shacking process. Moreover, the shacking process incorporated with plant fibers promoted and supported the mycotoxins degradation process.

## Graphical abstract

Milk contaminated with mycotoxins is a highly significant issue affecting human health. The study investigated the role of the shacking process on plant fibers binding process. The shacking process with plant fibers promoted and supported the mycotoxins degradation process.

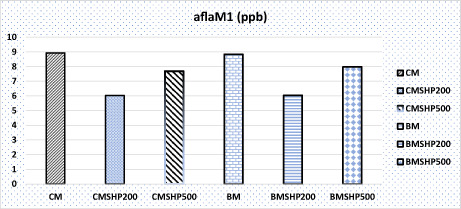

## 1 INTRODUCTION

Milk is a colloidal suspension that contains emulsified globules of fat, proteins, carbohydrate, lactose, minerals and vitamins. It is also a rich source of protective agents such as antibodies, enzymes and growth factors. Animal species, genetics, physiological status, the environment, and managerial considerations or handling procedures all affect the composition of milk in different ways. For instance, there are significant differences between the composition and characteristics of fresh cow's milk and buffalo milk. Buffalo's milk has a higher level of fat (7.64%), total protein (4.36%), total solids (19.3%), lactose (5.0%) and ash (0.76%) than cow's milk (4.9%, 3.7%, 14.1%, 4.8% and 0.65%, respectively) (Akinyemi et al., 2022).

When animals consume feed laden with mycotoxins due to the infestation of feed constituents (e.g., seeds and grains) by toxigenic molds, the milk can become contaminated with mycotoxins (Akinyemi et al., 2022). Mycotoxins in milk are a toxic secondary metabolite produced by some specific species of filamentous fungi, mainly belonging to the genera of *Aspergillus*, *Penicillium*, and *Fusarium*, which invade crops in fields and during storage (Akinyemi et al., 2022; Benkerroum, 2016). The European Commission recommended that the level of aflatoxin M1 (aflaM1) in raw milk should not exceed 50 ng/kg for adults and 25 ng/kg in infant milk (Omar, 2016). Studies showed that human consumption of contaminated milk by mycotoxins, particularly the more toxic parent forms, may lead to adverse effects on health, comprising growth groggy in children, toxicities of different tissues like the immune system, and finally causing cancer or death (Labuda & Tancinova, 2006; Rodrigues et al., 2011; Thukral et al., 2022).

Several approaches have been used to degrade mycotoxins in the contaminated dairy products, including milk (Benkerroum, 2016). Recently, the use of phenolic compounds in this sense is recognized due to their potential antioxidant properties. They are widely recommended as a safe, applicable and more efficient method than other synthetic agents (Abdelnaby et al., 2022; Aladaileh et al., 2020; Ismail et al., 2021; Pani et al., 2014; Youssef, Qari, Behiry, et al., 2021; Youssef, Qari, Matar, et al., 2021). The antioxidant capacity of phenolic compounds might be due to electron donation and hydrogen atom transfer to free radicals (Dai & Mumper, 2010) and chelated trace metals involved in radical development (Ghasemzadeh & Ghasemzadeh, 2011). *Camellia sinencese* is commonly recognized as green tea. It is mostly cultivated worldwide. It contains many active compounds that exhibit several biological activities, including antifungal, antibacterial, and antioxidant (Al‐Nemi et al., 2022; Blanco et al., 2003; Park et al., 2011; Saeed et al., 2020).

Another medicinal plant is *Hyphaene thebaica* (doum (DOU) epicarp fruit), which comprises a substantial amount of saponins, coumarins, hydroxycinnamates, essential oils and flavonoids (Sharaf et al., 1972). It also has nutritional trace minerals, proteins and fatty acids, in particular linoleic acid. In the previous studies, DOU extracts exhibited robust antimicrobial actions against gram‐positive and gram‐negative bacteria and a wide range of fungal isolates (Irobi & Adedayo, 1999; Mohamed et al., 2010; Salah et al., 2011). Additionally, licorice root (*Glycyrrhiza glabra*) is a perennial herbaceous plant used as a medicinal sweet drink by ancient Egyptian Pharaohs. It contains flavonoids (liquirtin, isoflavonoids and formononetin), saponin triterpenes (glycyrrhizin, glycyrrhetinic acid and liquirtic acid) (Abdelnour et al., 2020), and other constituents such as coumarins, amino acids, sugars, starch, tannins, choline, phytosterols and bitter principles (Arystanova et al., 2001; Ferreira et al., 2013; Pietri et al., 2010). Some scientific papers demonstrated the antifungal activity of *G. glabra* (Reddy et al., 2009), and its methanolic extract had high fungicidal effects against several pathogenic bacteria, including *Arthrinium sacchari* and *Chaetomium funicola* (Hojo & Sato, 2002; Saeed, Abd El‐Hack, et al., 2017).

There is very limited information about the plausible elimination of mycotoxins in buffalo or cow milk samples by DOU, green tea, and licorice root based on the literature. Furthermore, Irani et al. (2010) observed the inhibitory actions of the *G. glabra* extract on *Candida albicans* and some Gram‐positive bacteria.

Therefore, this study investigated, besides the fumonisins and other mycotoxins contaminated milk, the dual effects of the well‐ground plant parts as adsorbent(s) agents that bind the mycotoxin particles. Also, this study tested the effect of shacking process and its speed on the efficiency of the adsorption and binding process of mycotoxins and the new taste of the treated milk.

## 2 MATERIALS AND METHODS

### 2.1 Milk samples collection

Sixty‐four samples of unheated raw milk (2 kg each), where buffalo's milk (16 samples) and cow milk (48 samples) were obtained once per week from farmer's vendors at four open‐air rural big markets in west Alexandria (from Dekhila, Ameria, El Nasria and Borg El‐Arab regions). The samples were collected from November 2021 to January 2022. Each sample was packed in polyethylene bags and immediately kept under freezing conditions (−10°C) until analyzed (Ullah et al., 2020).

### 2.2 Samples used in the study

Dairy cattle milk was contaminated after cattle feeding with feeds contaminated with mycotoxins. Aflatoxins and fumonisins are considered the most common feed contaminants in maize feeds (De Nardi et al., 2014; Di Gregorio et al., 2014). Mycotoxins naturally contaminated the collected milk samples. After identifying mycotoxin concentrations and frequency percentages, two milk samples are taken to be used in this study. The selected samples were based on the number of mycotoxins contaminated in the sample and their concentrations. The first sample was chosen (cow's milk) was brought from a vendor (WFV) at Bourg El Arab rural market (Gomaa market). In contrast, the second sample (buffalo's milk) was brought from a vendor (WFV) at El Ameria rural (Talat) market.

### 2.3 Mycotoxins detection

The multi‐screening of mycotoxins was carried out in all collected samples using HPLC (Thermo Scientific Vanquish, Inc.) (NYSE: TMO) coupled with a Mass spectrometer (HPLC–MS/MS Pacific Biolab) (Rocchetti et al., 2021).

### 2.4 Determination frequency of mycotoxins naturally contaminated in the collected samples

The HPLC methods evaluated all the selected samples to determine the frequency of mycotoxin contamination (Fernández‐Cruz et al., 2010; Santos Pereira et al., 2019). All the determined mycotoxins were registered, and the frequency of each mycotoxin was calculated and registered as shown in Table 1. The selection of milk samples for the detoxification experiment was based on containing the highest number of mycotoxins and mycotoxins levels. The selected cow's milk samples were naturally contaminated with Aflatoxin M1 (aflaM1), cyclopiazonic acid (CPA), Zearalenone (Zear) and Diacetoxyscirpenol (Dias). Further, the selected buffalo's milk samples were naturally contaminated with aflaM1, CPA, Zear and Deoxynivalenol (Don).

**TABLE 1:** The contamination and occurrence levels of several mycotoxins in buffalo's and cow's milk samples.

Mycotoxin type	The frequency of milk samples (*n* = 64)		Mycotoxin levels (ppb)
	Buffalo milk (*n* = 16)	Cow milk (*n* = 48)	
Aflatoxin M_1_	62.5%	83.34%	14.64; 6.67 ± 1.08; 2.160 ± 0.66; 0.15 ± 0.450; 0.290 ± 0.154; 2.89 ± 0.180; 2.250 ± 0.350; 1.44 ± 0.55; 0.960 ± 0.520 0.48 ± 0.41; 0.120 ± 0.110; 0.0
Fumonisin	25%	None	11.24 ± 0.130; 6.053 ± 3.20; 3.24 ± 0.36; 0.0
Cyclopiazonic	43.75%	20.834%	10 ± 3.60; 10 ± 0.40; 2.24 ± 1.60; 1.98 ± 0.22; 0.210 ± 0.144; 0.41 ± 0.40; 0.0
Diacetoxyscrepinol	6.25%	14.584%	5 ± 0.018; 1.8 ± 0.023; 3.24 ± 0.011; 0.0
Deoxynevalenol	43.75	10.4167	1.44 ± 0.015; 0.576 ± 0.0140; 0.165 ± 0.505; 0.40 ± 0.02; 0.0
Zearalenone	56.25%	79.17%	7.63 ± 0.101; 4.55 ± 0.279; 4.789 ± 0.568; 3.769 ± 3.55; 4.339 ± 0.41; 2.28 ± 0.50; 1.02 ± 0.456

*Note*: None, not detected.

### 2.5 Plant collection, preparation and determination of active phenolic components

Plant parts (DOU, licorice and green tea) were brought from Ebn Sina medicinal plant market after estimation of any mycotoxin's contamination occurrence using multi‐screening TLC method according to (Fernández‐Cruz et al., 2010; Frisvad, 1995). All plant parts free of mycotoxins were used for our experiment. All selected parts of plants were sterilized with alcohol 70%, then oven dried at 40°C for 2 h to remove any alcohol traces to keep all components safe from degradation (Youssef, Qari, Behiry, et al., 2021; Youssef, Qari, Matar, et al., 2021). Afterward, the selected plant parts were finely ground in a sterilized electric grinder and then passed through a sterilized sieve with a mean pore size of 50.4 μm ± 0.9 to keep similar plant fibers. All the fine plant parts were frozen in sterilized plastic bags until utilization.

The extraction of phenolic compounds process was carried out using a GC (Agilent Tecnology7890A) coupled with a mass selective detector (MSD, Agilent7000 Triple Quad) equipped with Agilent HP‐5 ms capillary column. Apparatus (GC/MS/MS) made in (Germany) in Mass spectrophotometer Lab, at RCFF Cairo, Egypt, according to (Amorim et al., 2012; Saeed, Abd El‐Hack, et al., 2017). Briefly, fine powder (1 g, dry weight) from different tested plant parts and 4 mL of 80% methanol was mixed (at 60°C for 6 h) to obtain the methanolic extraction of medicinal plants. This extraction procedure was repeated three times. Then, extracts were centrifuged at 5000 *g* for 10 min and supernatants were collected for further analysis. The identification of components was based on comparing their mass spectra and retention time with those of the authentic compounds and by computer matching with NIST and WILEY library, as well as comparing the fragmentation pattern of the mass spectral data with those reported in the literature (Abdelkhalek et al., 2020; Youssef, Qari, Behiry, et al., 2021; Youssef, Qari, Matar, et al., 2021).

### 2.6 Detoxification experiment

The experiment was carried out by using plant parts finely ground as mycotoxins molecules bio‐binders in three ratios, 0.5%, 1% and 2%, in two steps as follows:

#### 2.6.1 1st step: Effect of bio binders in reducing mycotoxins without shacking process

The collected mycotoxins naturally contaminated milk samples (cow and buffalo(es) were used. Samples (100 mL each) were taken in a conical flask of 250 mL. The ground plant parts were added individually in three replicates at three concentrations of 0.5%, 1% and 2%. All samples were gently homogenized and kept overnight at 10°C. At the end of the incubation time, all samples were filtered by Watman filter paper (*n* = 1) then the filtrated samples were taken for mycotoxins estimation. The control samples (without any addition) were used for mycotoxins analysis using HPLC coupled with mass spectrometry according to (Rocchetti et al., 2021).

#### 2.6.2 2nd step: Effect of bio‐binders in reducing mycotoxins with shacking process

The samples were prepared as mentioned in the first step, but the samples with and without plant parts addition were shacked for 1 h at speeds 2 and 5 (200 and 500 rpm), respectively. At the end of the shacking process, the samples were filtered again by Watman (*n* = 1) filter paper. The supernatants were taken for multi‐mycotoxins estimation using HPLC coupled with mass spectrometry according to (Rocchetti et al., 2021), with certain modifications. The efficacy ratio of the mycotoxin binding process was calculated on the amount of residue of mycotoxin in milk samples after treatment with plant fiber and then filtration (Faucet‐Marquis et al., 2014; Peng et al., 2020).

### 2.7 Milk taste testing after plant fibers addition

Sterilized milk samples free of mycotoxins were prepared, each tested plant part was added and all milk samples with and without plant parts addition at 2% concentration were kept. The taste of milk after plant part addition at 2% was tested. All milk samples with 2% plant parts concentration and without addition were kept in the refrigerator overnight at 10°C. At the end of the incubation time, the treated milk samples were filtered via a clean sterilized cloth sheet then the taste was tested by three groups of 10 persons each. People who tasted these samples were among 30–40 years, healthy, males and live at the Alexandria Province.

### 2.8 Statistical analysis

A shapiro test was used in order to check for normality. Data were statistical analysis by one way ANOVA and the differences between means were detected via LSD test using CoStat program version 6.303 (CoHort software). The milk experiments were performed in triplicate in a completely randomized design with four variables (Duncan, 1955; McDonald, 2009).

## 3 RESULTS AND DISCUSSION

### 3.1 Frequencies of the determined mycotoxins naturally contaminated the collected samples

The frequency of each mycotoxin in the collected milk samples was calculated after multi‐screening the mycotoxins in naturally contaminated milk samples. Table 1 illustrated that aflatoxin M_1_ (aflaM_1_) was the highest contaminant in both buffalo and cow milk samples, with 62.5% and 83.34% contamination ratios, followed by Zear 56% and 79.17% for buffalo and cow milk samples, respectively. The frequency of CPA and Don was similar (43.75%) in buffalo milk samples, while they were 20.83% and 10.42% in cow milk samples, respectively. Conversely, the total fumonisins were not found in the tested cow milk samples, but it was 25% in buffalo milk samples. Animals that have consumed feeds contaminated with aflaM1, or other types such as Zear and CPA will be formed due to the metabolic process in the liver of ruminants and excreted in milk (Becker‐Algeri et al., 2016; De Nardi et al., 2014; Viegas et al., 2020). When consumed, this contaminated milk poses huge risks to humans, especially children. Also, animal feed's differing mycotoxin burdens could be considered the main factor affecting milk quality (Benkerroum, 2016). Our findings align with Viegas et al. (2020), who reported that dairy farming feed is contaminated with mycotoxins, affecting milk quality. The present data revealed that Dias was the lowest contaminant of the collected milk samples which realized occurrence ratios of 6.25% and 14.58% in buffalo and cow milk samples, respectively. Dias is a kind of mycotoxin with a low molecular weight produced by several Fusarium species (mainly *F. sambucinum*, *F. poae*, and *F. langsethiae*) (Knutsen et al., 2018). Furthermore, Dias has been identified in cereal grains and items made from cereal. Numerous investigations (Becker‐Algeri et al., 2016, 2020; Hof, 2016; Sirma et al., 2018; Thukral et al., 2022) have demonstrated that aflaM1 and Zear levels were the greater contaminants of milk samples, whereas CPA and ochratoxin levels were the lower contaminants.

### 3.2 Determination of active phenolic components in the tested plant parts

Table 2 lists the various phenolic compounds found in each of the medicinal plants that were put to the test. According to earlier scientific findings, the majority of the phenolic compounds found demonstrated antibacterial capabilities that helped to prevent the establishment and development of mycotoxins (Gauthier et al., 2016; Pani et al., 2014). Furthermore, compared to the standard group, the naturally occurring hydroxylated biphenyl and phenol types have a significant potential to reduce 3‐acetyl‐4‐deoxynivalenol synthesis by over 70% (Pani et al., 2014). The possible use of bioactive metabolites, such as phenolic compounds, which influence the growth and toxin production in the primary mycotoxigenic fungi has been described by a number of authors (Benbettaïeb et al., 2019; Benkerroum, 2016; Bhat & Al‐Daihan, 2014). According to Messier and Grenier (2011), licorice contains a variety of active chemicals that have antifungal properties. According to one study, licorice extract greatly lessened aflatoxin‐harmful B1's effects on chickens (Rashidi et al., 2020). It was documented that the highly antimicrobial potency of licorice was detected and its important role as an antifungal agent (Delbò, 2013; Park et al., 2011). The use of phytochemical plants as antifungal and antimicrobial agents has been previously described (Chen et al., 2020; Upadhyay et al., 2015). These mechanisms are associated with the high content of a wide array of active phenolic contents (Park et al., 2011; Saeed, Naveed, et al., 2017). Based on the literature, the phenolic contents of these plants have phytochemical chemoprotective constituents which possess a pharmacological effect such as hepato‐protective, immunologic and antimicrobial activities (Messier & Grenier, 2011).

**TABLE 2:** The main active phenolic components detected in tested medicinal plants (Green Tea leaves [GTL], Doum [DOU] epicarp, and licorice [LIC]).

Phenolic compounds of medicinal plants	Area sum %	Phenolic compounds medicinal plants	Area sum %
**Green tea leaves**			
Phenol,4‐(2‐aminopropyl)	11.6	5‐(2‐Aminopropyl)‐2‐methylphenol	6.0
Epigallo catechin gallate	26. 7	Flavonols	4.0
Epigallocatechin	16.6	Caffeine	7.5
3‐Phenyl‐2H‐chromene	7.95	Glutamic acid	1.26
Epicatechin gallate	2.61	Arginine	1.7
Theanine	6.10	Total	100
3‐[1‐Hydroxy‐2‐(methylamino) ethyl]phenol	7.98		
**Doum epicarp**			
p‐Allylphenol	2.19	Levoglucosenone	1.93
Ethylnorbornane	6.02	Octahyrochromen‐2‐one	2.2
Phenylglyoxylic acid	1.44	N‐Acetylneuraminic acid	1.93
2,5‐Diethyl‐para‐anisalehyde	9. 32	d‐Mannos	2.46
Sorbitol	0.83	2,6‐Dihydroxycineol	8.61
7,8‐ihydro‐α‐ionone	1.3	3,5‐Dihydroxyphenol	9.45
4‐Ethylbenzaldehye	3.09	Methyl 17‐methyloctadecanoate	1.16
Dimethoxyurene	0.54	3,4,7‐trimethylquerecetin	1.06
Ledol	1.22	Octadecanoic acid	8.47
Scopoletin	0.54	Glycitein	10.21
Farnesol	2.15	Luteolin 6,8‐C‐diglucoside	0.99
Hexestrol	0.8	Linoleic acid	14.49
Steviosie	2.37	5β,7βH,10α‐Eudesm‐11‐en‐1α‐ol	2.31
6‐Hyroxyflavone	1.65	Zeaxanthin	1.36
d‐Mannos	2.46	Total	100.00
**Licorice (*G. glabra*)**			
2,6‐Dimethylbenzaldhyde	0.81	Nabilone	0.92
p‐Allylphenol	2.32	Fisetin	0.93
Cedrol	2.32	Kaempferol	2.97
4‐methylactechol	2.81	5,7,3,4‐Tetrahydroxyflavanone	1.98
7,8‐Dihydro‐α‐ionone	2.35	β‐Patchoulene	1.99
Α‐Bisabolol	2.67	β‐Gurjunene	1.99
Dimethylcaffeic acid	2.67	ϒ‐Murolene	1.79
Scopoletin	2.40	Alloaromadendrene	1.35
Farnesol	2.41	α‐Himachalene	1.41
(−)‐β‐Pinene	2.42	Longipinene	1.47
Hexestrol	3.45	L‐Citrulline	1.52
Α‐Terpineol	3.45	Coumarin‐3‐carbonitrile,6‐methyl	1.54
ϒ‐Terpinene	3.47	Octadecanoic acid	1.54
6‐Hydroxyflavone	3.47	Stearic acid	2.81
Β‐Linalool	3.52	Luteolin 6,8,‐C‐diglucoside	2.99
Camphor	3.56	Linoleic acid	5.29
Chromone,5‐hydroxy‐6,7,8trimethoxy‐2,3‐dimethyl	3.59	Citronellic acid	5.73
2‐Allyl‐4‐methylphenol	2.62	Quinine	6.61
Isobornyl acetate	2.87	Total	100.00

### 3.3 Detoxifications using bio‐binders plant sources with and without shacking

The procedure of eliminating mycotoxins was carried out by simultaneously using finely powdered plant parts as adsorbent agents, bio‐binders, and biodegradation agents. After soaking and/or shacking treatments, the mycotoxins levels were assessed. Tables 3 and 4 display the results, which showed that cow milk with and without shacking was contaminated with mycotoxins when several bio‐binders' agents (green tea, DOU, and licorice) were used. The findings, as displayed in Table 3 and tableures 1, 2, 3, and 4, are related to the way mycotoxins attach to cow milk.

**TABLE 3:** Effect of licorice (LIC), doum (DOU) and green tea leaves (GTL) as bio binders on mycotoxins contaminated cow milk with and without shacking process.

Treatments^1^	Levels (%)	Mycotoxins levels (ppb)^2^				The ERs (%)^3^			
		aflaM1	CPA	Zear	Dias	aflaM1	CPA	Zear	Dias
Cow milk (CON)	—	8.92^a^	10.05^a^	7.15^a^	6.8^a^	—	—	—	—
CON+LIC	0.5	5.7^d^	3.83^f^	4.64^d^	0.06^o^	36.1	61.89	35.10	91.03
CON+LIC	1	2.67^e^	0.66^q^	1.27^i^	0.0^p^	70.07	93.43	82.24	100
CON+LIC	2	0.74^i^	0.39^s^	0.32^mn^	0.61^j^	91.70	96.12	95.52	99.12
CON+DOU	0.5	0.22^no^	3.67^g^	0.67^m^	0.19^n^	97.53	63.48	90.63	97.20
CON+DOU	1	0.29^m^	2.14^i^	0.45^lm^	0.30^l^	96.75	78.71	93.71	95.59
CON+DOU	2	0.67^j^	2.73^h^	1.99^g^	0.98^g^	92.49	72.83	72.17	85.59
CON+GTL	0.5	0.64^j^	6.12^d^	0.0^o^	3.56^b^	92.82	39.10	100	47.65
CON+GTL	1	0.38^l^	5.44^e^	0.0^o^	1.2^f^	95.74	45.87	100	82.35
CON+GTL	2	0.19^op^	1.98^j^	0.38^lm^	1.89^e^	97.87	80.30	94.68	72.20
CON+SH200	—	6.63^c^	7.14^c^	5.36^c^	2.84^c^	25.67	28.95	25.03	58.23
CON+SH500	—	7.68^b^	7.51^b^	6.66^b^	2.04^d^	13.90	25.27	6.85	70
CON+LIC+SH200	0.5	1.48^g^	0.28^uv^	1.5^h^	0.31^l^	83.41	97.21	79.02	95.44
CON+LIC+SH200	1	0.17^p^	0.08^x^	0.0^o^	0.07^o^	98.09	99.20	100	98.97
CON+LIC+SH200	2	0.05^r^	0.0^y^	0.09^o^	0.0^p^	99.44	100	98.74	100
CON+LIC+SH500	0.5	0.19^op^	0.98^n^	0.53^lm^	0.78^g^	97.87	90.25	92.59	88.53
CON+LIC+SH500	1	0.29^m^	0.67^q^	1.15^i^	0.66^i^	96.75	93.34	83.92	90.29
CON+LIC+SH500	2	0.79^h^	0.18^v^	1.73^g^	0.32^l^	91.14	98.21	75.80	95.29
CON+DOU+SH200	0.5	0.18^p^	0.3^u^	0.34^mn^	0.43^k^	97.98	97.01	95.24	84.86
CON+DOU+SH200	1	0.17^p^	1.3^k^	0.0o	0.75^h^	98.09	87.06	100	88.97
CON+DOU+SH200	2	0.11^q^	0.81o	0.34^mn^	0.45^k^	98.77	91.94	95.24	93.38
CON+DOU+SH500	0.5	0.22^no^	1.63^m^	0.40^lm^	0.0^p^	97.53	83.78	94.40	100
CON+DOU+SH500	1	0.23^n^	1.05^l^	2.11^f^	0.41^k^	97.42	89.55	70.49	93.97
CON+DOU+SH500	2	0.54^k^	0.64^q^	3.64^e^	1.23^f^	93.95	93.63	49.09	81.91
CON+GTL+SH200	0.5	0.0^s^	0.79^op^	0.07^o^	0.30^l^	100	92.14	99.02	95.89
CON+GTL+SH200	1	0.0^s^	0.76^p^	0.0^o^	0.0^p^	100	92.44	100	100
CON+GTL+SH200	2	0.0^s^	0.26^w^	0.0^o^	0.0^p^	100	97.41	100	100
CON+GTL+SH500	0.5	0.24^n^	0.59^v^	0.0^o^	0.25^m^	97.31	94.13	100	96.32
CON+GTL+SH500	1	1.48^g^	0.34^t^	0.17^no^	0.0^p^	83.41	96.62	97.62	100
CON+GTL+SH500	2	2.04^f^	0.0^y^	0.84^j^	0.0^p^	77.13	100	88.25	100
LSD 0.05	—	0.033	0.033	0.158	0.044	—	—	—	—

^1^ CON+LIC (control + licorice), CON+DOU (control + doum), CON+GTL (control + green tea leaves), CON+SH200 and 500 (control + shaking speed 200 and 500 rpm, respectively), CON+LIC+SH200 and 500 (control + licorice + shaking speed 200 and 500, respectively), CON+DOU+SH200 and 500 rpm (control + doum + shaking speed 200 and 500 rpm, respectively), CON+GTL+SH200 and 500 (control + green tea leaves + shaking speed 200 and 500, respectively). The means with the same letters are not significant.

^2^ aflaM1 (aflatoxin M1), cyclopiazonic acid (CPA), Zearalenone (Zear), Diacetoxyscirpenol (Dias).

^3^ Efficacy ratios (ERs %) of binder of mycotoxins.

**TABLE 4:** Effect of licorice (LIC), doum (DOU) and green tea leaves (GTL) as bio‐binders on mycotoxins contaminated buffalo milk with and without shacking process.

Treatments^1^	Levels (%)	Mycotoxins levels (ppb)^2^				The ERs %^3^			
		aflaM1	CPA	Zear	Don	aflaM1	CPA	Zear	Don
Buffalo milk (CON)	—	8.83^a^	13.6^a^	11.26^a^	8.24^a^	—	—	—	—
CON+LIC	0.5	1.57^g^	3.37^d^	1.64^e^	1.54^f^	82.22	75.22	85.43	81.31
CON+LIC	1	1.14^l^	1.7^g^	0.90^i^	1.48^g^	87.09	87.5	92.01	82.04
CON+LIC	2	0.51^r^	1.53^i^	0.57^k^	0.61^k^	94.22	88.75	94.94	92.60
CON+DOU	0.5	1.40^i^	2.24^e^	1.08^h^	3.52^c^	84.14	83.53	90.41	57.28
CON+DOU	1	0.65^p^	0.31^p^	1.32^f^	0.39^n^	92.64	97.72	88.28	95.27
CON+DOU	2	0.16^y^	1.09^h^	0.78^j^	0.16st	98.19	91.98	93.07	98.06
CON+GTL	0.5	2.33^e^	1.66^g^	0.44^l^	0.98^i^	73.61	87.79	96.09	88.11
CON+GTL	1	2.08^f^	0.80^m^	1.19^g^	0.30^p^	76.44	94.12	89.43	96.36
CON+GTL	2	0.40^t^	0.48^o^	0.05^q^	0.22^qr^	95.47	96.47	99.55	97.33
SH200	—	6.04^c^	7.2^c^	7.4^b^	3.64^b^	31.60	47.09	34.28	55.82
SH500	—	7.98^b^	9.24^b^	7.02^c^	3.42^d^	9.63	32.59	37.65	58.49
CON+LIC+SH200	0.5	0.32^v^	0.94^l^	0.30^n^	1.32^g^	96.38	93.09	97.33	83.98
CON+LIC+SH200	1	0.39^t^	0.21^q^	0.20^o^	0.27^pq^	95.58	98.45	98.22	96.72
CON+LIC+SH200	2	0.98^n^	0.04^r^	0.0^r^	0.0^u^	88.90	99.70	100	100
CON+LIC+SH500	0.5	5.26^d^	0.21^q^	0.53^k^	1.92^e^	40.43	98.45	95.29	76.70
CON+LIC+SH500	1	1.30^j^	0.0^r^	1.29^f^	0.78^j^	82.28	100	88.54	90.53
CON+LIC+SH500	2	1.08^lm^	0.0^r^	1.09^h^	0.42^m^	87.77	100	90.08	94.90
CON+DOU+SH200	0.5	0.0^z^	1.92^f^	0.40^lm^	0.0^u^	100	85.88	96.45	100
CON+DOU+SH200	1	0.15^y^	1.03^h^	0.36^m^	0.34^no^	98.30	92.43	96.80	95.87
CON+DOU+SH200	2	0.45^s^	1.07^h^	0.0^r^	0.36^no^	94.90	92.13	100	95.63
CON+DOU+SH500	0.5	0.23^w^	0.54^no^	0.0^r^	0.20^rs^	97.39	96.03	100	97.57
CON+DOU+SH500	1	1.06^m^	0.0^r^	0.36^m^	0.40^mn^	87.99	100	96.80	95.14
CON+DOU+SH500	2	1.21^k^	0.34^p^	0.37^m^	0.96^i^	86.30	97.5	96.71	88.35
CON+GTL+SH200	0.5	1.46^h^	1.44^k^	0.14^p^	0.48^l^	83.46	89.41	98.76	94.17
CON+GTL+SH200	1	0.78^o^	0.59^n^	0.20^o^	0.25^pq^	91.17	95.66	98.22	96.97
CON+GTL+SH200	2	0.59^q^	0.83^m^	0.36^m^	0.14^t^	93.32	93.89	96.80	98.30
CON+GTL+SH500	0.5	0.16^y^	0.65^n^	1.71^d^	0.25^pq^	98.19	95.22	84.81	96.97
CON+GTL+SH500	1	0.19^x^	0.52^o^	0.37^m^	0.17st	97.85	96.18	96.71	97.94
CON+GTL+SH500	2	0.22^w^	0.37^p^	0.20^o^	0.27^pq^	97.51	98.38	98.22	96.72
L.S.D0.05	—	0.016	0.061	0.045	0.045	—	—	—	—

^1^ CON+LIC (control + licorice), CON+DOU (control + doum), CON+GTL (control + green tea leaves), SH200 and SH500 (shaking at 200 and 500 rpm, respectively), CON+LIC+SH200 and 500 (control + licorice + shaking speed 200 and 500, respectively), CON+DOU+SH200 and 500 rpm (control + doum + shaking speed 200 and 500 rpm, respectively), CON+GTL+SH200 and 500 (control + green tea leaves + shaking speed 200 and 500, respectively). The means with the same letters are not significant.

^2^ aflaM1 (aflatoxin M1), cyclopiazonic acid (CPA), Zearalenone (Zear), Deoxynivalenol (Don).

^3^ Efficacy ratios (ERs %) of binder of mycotoxins.

### 3.4 Soaking process

At a dose of 0.5%, DOU fibers showed the highest ratio in binding and degrading aflaM_1_ (97.53%) and CPA (6348%) with statistical differences, followed by the green tea leaves (GTL) treatment in binding or eliminating Zear (90.63%) from milk samples.

In this sense, GTL exhibited the best bio‐binders in degrading Zear (100%). Regarding the Dias, co‐treating with DOU presented the most effective bio‐binder and degrading agent for Dias (97.20%), followed by licorice (LIC) (91.03%). All the plant fibers used as bio‐binders in this trial exhibited significantly moderate efficacy in binding and degrading CPA at a dose of 0.5% (63.48% and 61.89%) for DOU and LIC, respectively.

At dose, 1%: DOU produced the best bio‐binder and degrading treatment in reducing aflaM_1_ with a ratio of 96.75%, followed by GTL (95.74%), but LIC was the best treatment in binding and degrading for CPA (93.43%) followed by DOU treatment (78.71%). All the tested bio‐binders and degrading treatments were exhibited significantly higher efficient in getting rid and removing Dias from milk. In this sense, medicinal plants' efficiency in removing the Dias in milk samples was 100%, 95.59% and 82.35% for LIC, DOU, and GTL, respectively. On the other hand, GTL was the best treatment for completely binding and degrading Zear (100%), followed by DOU (93.71%) then LIC (82.24%), respectively.

At the dose of 2%: the detoxification of aflaM1 rations in milk samples were 97.89%, 92.49% and 91.70% for GTL > DOU > LIC, successively. For the removal of CPA in milk samples, results indicate that LIC > GTL > DOU efficiencies were 96.12%, 80.30% and 72.83%, respectively. A similar trend was detected for Zear, whereas the best results were produced by LIC (95.52%), followed by GTL (94.68%) and then DOU (72.17%). For the Dias biodegradation, the efficacies of LIC > DOU > GTL were 91.03%, 85.59% and 72.20% respectively. Our data exhibited that the detoxification process using bio‐binding and biodegradation treatments was affected by the treatment's concentration, the bio binder's variety and its phenolic components content, and the type or structure of mycotoxin (Table 3).

### 3.5 Shacking process

**FIGURE 1:**
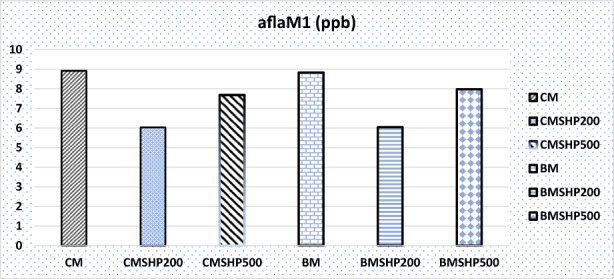
Effect of shacking speed on the binding process of aflatoxin M1 (aflaM1) in milk samples. BM, buffalo milk, BMSHP200, buffalo milk treated with shacking speed 200 rpm; BMSHP500, buffalo milk treated with shacking speed 500 rpm; CM, cow milk; CMSHP200, cow milk treated with shacking speed 200 rpm; CMSHP500, cow milk treated with shacking speed 500 rpm.

**FIGURE 2:**
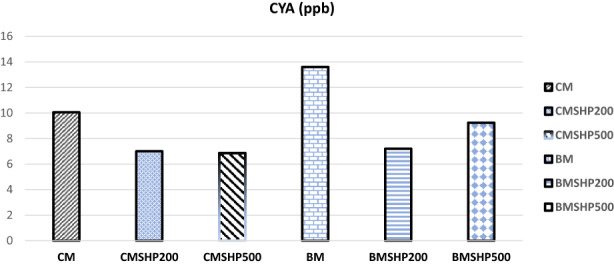
Effect of shacking speed on the binding process of cyclopiazonic acid (CYA, ppb) in milk samples. BM, buffalo milk, BMSHP200, buffalo milk treated with shacking speed 200 rpm; BMSHP500, buffalo milk treated with shacking speed 500 rpm; CM, cow milk; CMSHP200, cow milk treated with shacking speed 200 rpm; CMSHP500, cow milk treated with shacking speed 500 rpm.

**FIGURE 3:**
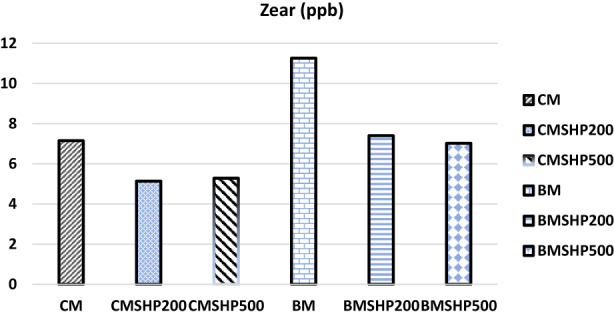
Effect of shacking speed on the binding process of zearalenone (Zear, ppb) in milk samples. BM, buffalo milk, BMSHP200, buffalo milk treated with shacking speed 200 rpm; BMSHP500, buffalo milk treated with shacking speed 500 rpm; CM, cow milk; CMSHP200, cow milk treated with shacking speed 200 rpm; CMSHP500, cow milk treated with shacking speed 500 rpm.

**FIGURE 4:**
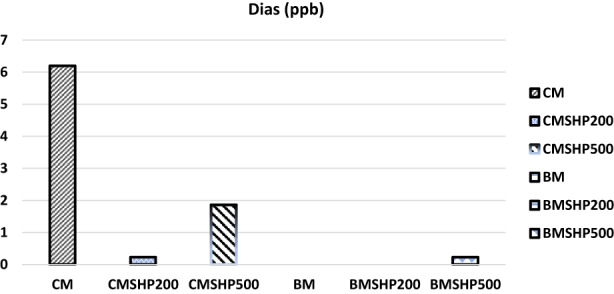
Effect of shacking speed on binding process of diacetoxyscirpenol (Dias, ppb) in milk samples. BM, buffalo milk, BMSHP200, buffalo milk treated with shacking speed 200 rpm; BMSHP500, buffalo milk treated with shacking speed 500 rpm; CM, cow milk; CMSHP200, cow milk treated with shacking speed 200 rpm; CMSHP500, cow milk treated with shacking speed 500 rpm.

**FIGURE 5:**
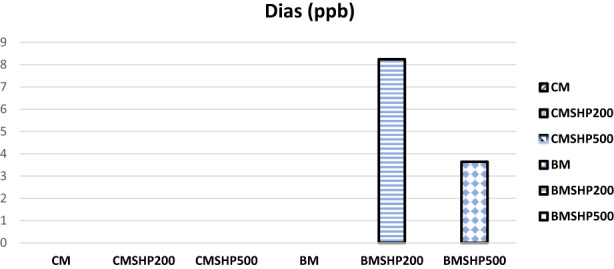
Effect of shacking speed on binding process of deoxynivalenol (Dias, ppb) in milk samples. BM, buffalo milk, BMSHP200, buffalo milk treated with shacking speed 200 rpm; BMSHP500, buffalo milk treated with shacking speed 500 rpm; CM, cow milk; CMSHP200, cow milk treated with shacking speed 200 rpm; CMSHP500, cow milk treated with shacking speed 500 rpm.

The shacking and filtration processes of milk samples have shown certain moderate effects in degrading mycotoxins (without any plant fiber addition) except in the Dias case (Figures 1, 2, 3, 4, 5). Significant reductions of all mycotoxins were detected by the GTL (0.5%, 1% and 2%).

At shacking speed of 200 rpm (SH200) for 1 h, the reduced concentrations of each mycotoxin were as the following: aflaM1 (25.67%), CPA (28.95%), Zear (25.03%) and Dias (58.23%). All the mycotoxins studied (except Dias) were decreased after the treating shacking process at the speed 500 rpm (SH500) as follows: aflaM_1_ (13.90%), CPA (25.27%), Zear (6.85%) and Dias (70%). After that, increasing the shacking speed by more than 200 rpm might reduce the efficiency of biodegradation of mycotoxins. This variation in mycotoxin behavior as a response to different shaking speeds could be associated with the differences in their molecule's chemical and physical properties and molecular weights (Omar, 2016). This notice matched those of (Youssef, 2019a), who reported that mycotoxins particles have differed in their dimensions. Furthermore, the molecular weight of each tested mycotoxin molecules according to (https://pubchem.ncbi.nlm.nih.gov/compound/54682463) were as follows: Dias (366 g/mol) > CPA (336.4 g/mol) > aflaM1 (328.27 g/mol) > Zear (318 g/mol) > Don (296.31 g/mol).

At a concentration of 0.5%, our present study showed that GTL was the best treatment in binding and removing all the occurred mycotoxins as shown in the following order aflaM1 (100%), Zear (99.02%), Dias (95.89%) and CPA (92.14%) followed by DOU which realized efficacy ratios in the following order: aflaM1 (97.98%), CPA (97.01%), Zear (95.24%) and Dias (84.86%). Moreover, the application of LIC fibers treatment was not very efficient as a mycotoxin bio‐binder and biodegraded agent for Zear and aflaM1 as follows: CPA (97.21%), Dias (95.44%), aflaM1 (83.41%), and Zear (79.02%). The outcomes of this trial are consistent with other research (Upadhyay et al., 2015) and (Youssef, Qari, Behiry, et al., 2021; Youssef, Qari, Matar, et al., 2021) showed mycotoxins like aflatoxin and Zear can be significantly inhibited by plant derivatives such DOU, GTL, banana peels, and LIC.

At concentrations of 1% and 2%, GTL was still the best treatment in removing all the mycotoxins, which realized the following efficacies at levels 1% and 2%: for aflaM1, Zear and Dias (100%). CPA has reduced with 92.44% and 97.41% efficacies at 1% and 2% concentrations, respectively. At the same time, LIC was the second‐best treatment which realized at a concentration of 1% the following removing efficacy ratios for Zear (100%), CPA (99.20%), Dias (98.90%) and aflaM_1_ (98.09%). Furthermore, these efficacy ratios were augmented with licorice 2% to achieve the following ratios: 100% for both CPA and Dias, which possess a higher molecular weight (https://pubchem.ncbi.nlm.nih.gov/compound/54682463), then (99.44%) for aflaM1. On the other hand, the removal efficacy ratio of Zear dropped at concentrations of 2 percent of both LIC and DOU from 100 percent (at 1 percent concentration) to 98.74 percent and 95.24 percent, respectively, which may be associated to an imbalance enhancement between the constituents of this employed bio‐binder and their related substituents at high concentrations (Hatano et al., 2017). Our results also showed that not all mycotoxins responded to the same therapy in the same way. In line with the data obtained in this experiment, several studies revealed that citrinin, ochratoxin, alernariol monomethyl ether, and tenuazonic acid do not have the same inhibition response against the same plant powder or extract (Youssef, 2019b; Youssef, Qari, Behiry, et al., 2021). In line with those results, Moussa (Moussa et al., 2020) and Intanoo (Intanoo et al., 2020) reported that several types of clay potentially remove aflaM1 in contaminated milk.

At shacking speed 500 rpm: In case of LIC, the efficacy ratio (ER %) of this bio‐binder in adsorbing and degrading mycotoxins was as follows: At concentration 0.5%, aflaM1 (97.87%) > Zear (92.59%) > CPA (90.25%) > Dias (88.53%). At concentration 1%, aflaM1 (97.75%) > CPA (93.34%) > Dias (90.29%) > Zear (83.92%). At concentration 2%, CPA (98.21%) > Dias (95.29%) > aflaM1 (91.14%) > Zear (75.80%). In all cases, our results illustrated that LIC was very effective in removing all the mycotoxins naturally contaminated cow milk samples, specially aflaM1, at all the tested concentrations. Our data followed those of (Mohseni et al., 2014; Rashidi et al., 2020), who reported that LIC has antitoxin effects and can completely inhibit aflatoxin B1 at a concentration of 10 g/mL.

In the case of DOU (0.5%), the ER % of DOU in removing the following toxins were as the following, Dias (100%) > AflaM1 (97.53%) > Zear (94.40%) > CPA (83.78%). Whereas at concentration 1%, AflaM1 > Dias (93.97%) > CPA (89.55%) > Zear (70.49%). But at concentration 2% AflaM1 (93.95%) > CPA (93.63%) > Dias (81.91%) > Zear (49.09%). Similar to our data, (Youssef, Qari, Behiry, et al., 2021; Youssef, Qari, Matar, et al., 2021) reported the capability of DOU in inhibiting aflatoxin B_1_. Additionally, green leafy vegetables have moderate antibacterial activity (Bhat & Al‐Daihan, 2014). Those medicinal plants have various pharmacologically active compounds and thus provide the scientific basis for the traditional uses of the considered medicinal plant in treating contaminated milk.

In the case of GTL, it was very effective in removing and degrading all the targeted mycotoxins where its efficacy ratios are in the following order: at concentration 0.5% Zear (100%) > aflaM1 (97.31%) > Dias (96.32%) > CPA (94.13%). While, at concentration, 1%, the efficacy ratios order is: Dias (100%) > Zear (97.62%) > CPA (96.62%) > aflaM1 (83.41%). On the other hand, at the concentration of 2%, the efficacy ratios were changed to the following order: CPA and Dias (100%) > Zear (88.25%) > aflaM1 (77.13%). Our findings were globally matched with the study of (Saeed et al., 2018), who mentioned that GTL has several antioxidant components such as catechin, epicatechin, gallocatechin, epigallocatechin and epigallocatechin gallate. These active components in GTL are considered very effective chemo‐preventive agents against both natural and chemical toxins (Saeed et al., 2018; Youssef, 2019a). Moreover, green tea is rich in catechin epigallocatechin gallate (EGCG) at 0.25%, effectively decreasing aflatoxin production by reducing the expression of aflS and aflR (Phillips et al., 2008; Xu et al., 2021).

### 3.6 Buffalo milk sample

The buffalo milk sample has the same mycotoxins that infected the cow milk, except that Don is present instead of diacetoxyscirpenol in the cow milk tested sample, as shown by our results in Table 4 and Figures 1, 2, 3 and 5.

### 3.7 Effects of the soaking process

The efficacy ratios (ERs %) of LIC in removing mycotoxins are presented in Table 3. Their efficacy ratios were ascendant (2% > 1% > 0.5%) for all the types of the mycotoxins contaminants as illustrated in the following order: Zear (94.94%) > aflaM_1_ (94.22%) > Don (92.60%) > CPA (88.75%). Otherwise, the behavior of DOU in removing mycotoxins from milk samples differed depending on their concentrations. The efficacy ratios of DOU in removing mycotoxins were the best at a concentration of 2% for all the mycotoxins contaminants, but 1% was the best for removing CPA. At the same time, the worth efficacy ratios (ERs %) were 57.28% and 83.53% for both Don and aflaM1, respectively, at 0.5% and 88.25% for Zear at 1%. The order of the efficacy ratios was as follows: aflaM1 (98.19%) > Don (98.06%) > CPA (97.72%) > Zear (93.03%). The behavior of GTL in removing mycotoxins was ascendant and proportional to its concentration in all the mycotoxins contaminants except in the Zear case. At a concentration of 5%, the best‐removed mycotoxin ratio was detected for Zear (96.09%), followed by Don (88.11%). At the concentration of 1% of GTL, Don then CPA were the higher removed mycotoxins with ERs % 96.36% and 94.12%, successively. On the other hand, the best concentration in removing mycotoxins was detected at a concentration of 2% as the following order: Zear (99.55%) > Don (97.33%) > CPA (96.47%) > aflaM1 (95.47%). Overall, our results illustrated that GTL showed the best efficacy ratio in removing Zear (99.55%) and better in removing Don, CPA and aflaM1 from cow milk samples. Our results agree with (Intanoo et al., 2020; Upadhyay et al., 2015), who reported that polyphenol extracted from GTL presented substantial inhibition for the production of both Zear and Don. Furthermore, DOU was the best removal agent of aflaM1, CPA and Don. Our results coincided with those of (Youssef, Qari, Behiry, et al., 2021). The high efficiency of DOU as a mycotoxins bio‐binder may be due to its richness in phenolic compounds, vitamins, and fibers, as a good adsorbent agent of mycotoxins. Moreover, the mycotoxins binder of DOU could be supported by its high content of fibers (Aboshora et al., 2014).

### 3.8 Effects of shacking process

Our results exhibited that shacking process and then filtration moderately induced the mycotoxins degradation process, which was augmented by increasing the shacking speed with Zear and Don or decreased as augmented speed such as aflaM1 and CPA.

Shacking speed at 200 rpm (SH200): In the case of LIC, the concentration of 2% realized the highest efficacy ratios in binding CPA (99.70%) and entirely binding both Zear and Don (100%). The efficacy of removing aflaM1 in buffalo milk samples was 96.38% and 95.58% for 0.5% and 1.0%, respectively.

In the case of DOU, 0.5% of DOU exhibited degradation of both aflaM1 and Don completely, while Zear was entirely removed by 2% treatment and the CPA was highly removed at a concentration of 1% with an efficacy ratio (92.43%).

In the case of GTL, the ERs were 93.32% and 98.30% for aflaM1 and Don at 2% treatment, respectively. Furthermore, the CPA (95.66%) and Zear (98.76%) were highly removed by 1% and 0.5%, respectively. The data obtained revealed that the bio‐binder's efficacy ratio depended on the concentration of the used plant and the type of mycotoxins. Our results are in harmony with Becker‐Algeri et al. (2016) and Ismail et al. (2021). Those previous works indicated that the efficacy of plant extracts depends on their varieties, quantities and components of a particular plant extract. Its antioxidants reduce aflatoxins by adsorbing, neutralizing the free radicals, preventing their proliferation chains, and resulting in less hazardous compounds. The present study also coincided with those of (Benbettaïeb et al., 2019; Youssef, 2019a), who reported that the active compounds of the same plant part varied in their modes of action according to their concentration and the nature of the tested microorganism.

Shacking speed at 500 rpm (SH500): In the case of LIC, the removal process was ascendant according to the augmented concentrations in the case of aflaM1, CPA and Don but descendant in the case of Zear. The best ER % in the case of aflaM1 and Don was at 2% (87.77%) and (94.90%), respectively. But CPA was entirely removed in 1% and 2% concentrations. The best‐removed concertation of Zear (95.29%) was found at level 0.5%.

In the case of DOU, both aflaM1 and Don were highly removed at level 0.5% with ERs % (97.39%) and (97.57%) successively. At the same time, Zear and CPA were removed at 0.5% and 1% concentrations, respectively.

In the case of GTL, only aflaM1was highly removed at the level 0.5% with ERs % (98.19%), while at level 2%, GTL exhibited a highly effective tool in removing CPA and Zear with ERs % (98.38%) and (98.22%), respectively. Regarding Don, the ERs was 97.94% at a concentration of 1%.

All the present data indicated that both levels of GTL (0.5% and 1.0%) exhibited the best binder in removing aflaM1 and Don in buffaloes' milk samples, respectively. Moreover, CPA was entirely removed by LIC and DOU at level 1.0% and Zear which was completely removed at DOU 0.5%. The differences in mycotoxins responses to phyto‐ treatment levels might be due to the number of active compounds in each studied medicinal plant. Authors suggested that the isolation of several active compounds from those plants could lead more precisely to explain the potential role in removing the capacity of mycotoxins in milk. In this sense, our results were matched with those of (Peng et al., 2020; Youssef, 2019b), who mentioned that the different types of mycotoxins and the different producers of the same mycotoxin (Youssef, 2019a) differed in their action against the applied bio‐binder and its applied dose. Because plant fiber concentration was taken into account at numerous adsorption sites, it was previously shown (Adunphatcharaphon et al., 2020; Khaskheli et al., 2011) that the concentration of the adsorbent agent significantly impacts the adsorption process. It has been suggested that GTL polyphenols can control detoxification enzymes' activity, inhibit cancer, and prevent genetic alterations (Abd El‐Hack et al., 2020). Otherwise, according to Youssef, Qari, Behiry, et al. (2021), the same mycotoxin does not react similarly to applied plant treatments at various concentrations.

### 3.9 Effects of shacking speed process on the binding efficiency of the applied plant fibers in case of cow and buffalo milk samples

#### 3.9.1 Cow milk sample

As seen in Table 5, the following consequences are demonstrated: In the case of aflaM1, the inhibition of aflaM1 efficiency of cow milk sample with shacking process (SH200) without any plant fiber addition was approximately exhibited two fold higher than the inhibition at SH500. The synergistic effect between the applied shacking speed and the plant fiber types at different concentrations showed the best results with the SH200. At SH200, the GTL removed aflaM1 at all its tested levels, followed by LIC, then DOU at level 2% with efficacy ratios (99.24%) and (98.34%), respectively. Our results indicated that the shacking speed process produced an additive effect in binding aflaM1. This result is in parallel with the work of (Adunphatcharaphon et al., 2020; Saeed, Naveed, et al., 2017; Xu et al., 2021) that mentioned that the removal strategy for aflatoxin contamination by GTL was very effective due to its high content of catechin and EGCG. EGCG is one of the most active and richest molecules in GTL because it was reported as a vigorous antioxidant (Xu et al., 2021). Additionally, phytochemicals isolated from date pit can effectively adsorb mediators to eliminate aflaM1 and ochratoxins from contaminated milk (Abdelnaby et al., 2022).

**TABLE 5:** Effect of shacking speed process on the binding efficiency ratios (ERs %) of the applied plant fibers in cow milk sample.

Treatments^1^	Levels (%)	ERs (%)^2^							
		Shaking speed (200 rpm)				Shaking speed (500 rpm)			
		aflaM1	CPA	Zear	Dias	aflaM1	CPA	Zear	Dias
Cow milk (CON)	—	25.67	28.95	25.03	58.23	13.90	25.27	6.85	70
CON+LIC	0.5	57.73	96.08	72.01	89.08	97.53	86.95	92.04	61.76
CON+LIC	1	97.43	98.88	100	97.53	96.22	91.08	82.73	67.65
CON+LIC	2	99.24	100	98.32	100	89.71	97.60	74.02	84.31
CON+DOU	0.5	79.28	95.80	93.66	84.86	97.13	78.29	93.99	100
CON+DOU	1	97.43	88.65	100	73.59	97.00	86.02	68.32	39.71
CON+DOU	2	98.34	81.79	93.66	84.15	92.97	91.48	45.34	79.90
CON+GTL	0.5	100	88.93	98.69	89.44	96.87	92.14	100	87.74
CON+GTL	1	100	89.35	100	100	80.73	95.47	97.45	100
CON+GTL	2	100	96.36	100	100	73.44	100	87.39	100

^1^ CON+LIC (control + licorice), CON+DOU (control + doum), CON+GTL (control + green tea leaves).

^2^ Aflatoxin M1 (aflaM1), Cyclopiazonic acid (CPA), Zearalenone (Zear) and Diacetoxyscirpenol (Dias).

In the case of CPA, the binding efficiency of all the tested plant fibers augmented with the SH200 groups, especially in the case of LIC at level 2%, which entirely bound CPA followed by LIC 1% (98.88%), then GTL at 2% (96.36%). At the same time, the plant fibers' binding process efficiency decreased in SH500 groups except in the case of GTL at 2%, which entirely binding and removed the CPA. Our results indicated that the shacking speed process played a very important role combined with the plant fibers binding to eliminate the toxins from contaminated milk. Moreover, (Alam et al., 2022) reported that the shacking speed is an important factor influencing the adsorption or de‐adsorption of solutes from adsorbents (Asses et al., 2018). The adsorption process is controlled by several factors, including shacking speed, particle size, adsorbent concentration, shacking time, pH, and incubation temperature.

In the case of Zear, the SH200 group is better than SH500 for binding Zear with all the tested plant fibers, especially with both plants LIC and DOU at level 1% and GTL at levels 1% and 2% which entirely binding Zear followed by GTL at level 0.5% (98.96%) then LIC at level 2% (98.32%). Furthermore, the behavior of plant fibers (types and their different concentrations) in binding mycotoxin was completely changed according to the applied shacking speed, duration time, and the type of the mycotoxin (Table 5). The Zear binding process efficiency was ed with SH500 group, while it did not appear to affect aflaM1 and CPA eliminations.

We noticed that the Zear binding process efficiency of LIC, DOU and GTL at level 0.5% increased from 72.01%, 93.66% and 98.69% to 93.04%, 93.66%, and 100%, respectively. Our findings are endorsed by those of (Wang et al., 2020), who reported that the adsorption of Alternaria mycotoxins (AOH and AME) was partially reversible.

In the case of Dias, both LIC and GTL at level 2% and GTL at level 1% were entirely binding Dias with a shaking speed of 200 rpm. DOU (0.5%) and GTL (1% or 2%) completely could remove Dias with shacking speed 500 rpm. Plant fiber concentrations in the binding Dias process were still different from the other mycotoxins binding processes. Our findings have coincided with those of (Adunphatcharaphon et al., 2020; Luo et al., 2020), who reported that the adsorption of aflam1 and Zear was different from that of Don and Fumonisin according to the complex and the chemical bond made between the adsorbent polysaccharides and the mycotoxin which affected by its stability. Our results are highly endorsed by those (Aladaileh et al., 2020; Kulasooriya et al., 2020) who reported that the speed of shacking, the concentration of the adsorbent and the time of contact affected the adsorption process. They also reported that the best speed of shacking to get high binding capacity was 200 rpm.

Furthermore, our findings were highly in agreement with (Alam et al., 2022), who reported that the shacking speed controls the mass transfer parameter in the adsorption process, influencing the distribution of the adsorbent in the solution. They also mentioned that the rate of the adsorbed and removed dye was reduced upon the increase in the shacking speed. Our findings are also in harmony with those of (Adunphatcharaphon et al., 2020; Rasheed et al., 2020), who reported that toxin adsorption is not affected by the presence of one or multi different mycotoxins in the liquid medium but was affected by the pH and thermodynamic parameters, suggesting that mycotoxin adsorption is an exothermic and there was a hydrophobic interaction (like aflam1 and Zear) or polar noncovalent interaction (like Don) may be associated according to the adsorbed mycotoxin.

#### 3.9.2 Buffalo milk samples

Data shown in Table 6 illustrated the followings, only DOU (0.5%) at shack speed 200 rpm was entirely binding aflaM1 followed by 1% of DOU at shaking speed 200 (97.52%), then GTL addition with shaking speed 500 (97.99%, 97.62% and 97.24%) respectively. For CPA, the shaking speed of 500 rpm realized the best CPA binding ratio of 100% by using LIC (1% or 2%) and DOU (1%) followed by LIC (2%) at shacking speed of 500 (99.45%). Regarding Zear, LIC and DOU at level 2% are entirely adsorbed Zear particles with shacking speed of 200 rpm followed by GTL at level 0.5% with the same shacking speed. On the other hand, Zear was entirely bound with DOU (0.5%) with a shaking speed 500 rpm. In comparison, the adsorption efficacies were reduced at shacking speed 500 rpm (except 0.5% DOU and 2% GTL), which indicated that the same concentration of the same plant fibers type differed in its binding efficacy according to the applied shacking speed. Our results illustrated, as shown in Tables 5 and 6, that the same types of mycotoxins contaminating milk differed in their response to the adsorption process according to the type of milk, as they were more susceptible to being adsorbed in cow's milk than in buffalo milk with the same treatments under the same experimental conditions. This feature might be correlated with the differences in chemical compositions between cows' milk and buffaloes. Our results align with the data obtained by (Enb et al., 2009; Nazir et al., 2015; Oksa, 2019), who reported that buffalo milk was higher in fat, protein, ash, lactose and non‐solid fats than cow milk.

**TABLE 6:** Effect of shacking speed process on the binding efficiency ratios (ERs %) of the applied plant fibers in buffalo milk sample.

Treatments^1^	Levels (%)	ERs (%)^2^							
		Shaking speed (200 rpm)				Shaking speed (500 rpm)			
		aflaM1	CPA	Zear	Dias	aflaM1	CPA	Zear	Dias
Buffalo milk (CON)	—	31.60	47.09	34.28	55.82	9.63	32.59	37.65	58.49
CON+LIC	0.5	94.70	86.94	95.95	63.74	34.08	97.73	92.45	43.86
CON+LIC	1	93.54	97.08	97.30	92.58	83.71	100	81.62	77.19
CON+LIC	2	83.77	99.45	100	100	86.47	100	84.47	87.72
CON+DOU	0.5	100	73.34	94.59	100	97.12	94.15	100	94.15
CON+DOU	1	97.52	85.69	95.13	90.66	86.72	100	94.87	88.30
CON+DOU	2	92.55	85.14	100	90.11	84.84	96.32	94.73	71.93
CON+GTL	0.5	75.83	80.0	98.11	86.81	97.99	92.96	75.64	92.69
CON+GTL	1	87.09	91.80	97.30	93.13	97.62	94.37	94.73	95.03
CON+GTL	2	90.23	88.47	95.13	92.58	97.24	95.99	97.15	92.10

^1^ CON+LIC (control + licorice), CON+DOU (control + doum), CON+GTL (control + green tea leaves).

^2^ Aflatoxin M1 (aflaM1), Cyclopiazonic acid (CPA), Zearalenone (Zear) and Diacetoxyscirpenol (Dias).

The mode of action of the binding and adsorption process of mycotoxin particles onto each plant fiber can be explained by Di Gregorio et al. (2014). On the surface of the fiber, each mycotoxin particle has adsorbed. Each mycotoxin's particle size, the diameter and surface of the plant fiber, and the characteristics of each plant fiber impacted the adsorption process. The adsorption of aflam1 and Zear was not impacted by the pH of the medium, whereas Don's adsorption was particularly sensitive to pH change (Greco et al., 2019).

### 3.10 Tasting experiment of milk after plant fibers addition and filtration

This experiment tried to taste the modified milk after phytochemical additions. Three groups of people (10 in each group) were included in this test. People preferred the milk taste with LIC because it is sweet and almost like milk without any addition (Table 7). Moreover, the sweet, bitter, and any change in taste were tested rather than the normal milk taste. Table 7 shows that milk treated with DOU > Milk treated with LIC > with GTL. Regarding bitter taste, few people (about 2% of the three groups) declared that milk treated with GTL has a bitter taste. Change in taste than their people habits: Milk treated with LIC > milk treated with DOU > milk treated with GTL.

**TABLE 7:** Modified milk taste testing for consumers (10 people per group).

Treatment^1^	Levels (%)	Groups^2^			Average (%)
		Group 1 (%)	Group 2 (%)	Group 3 (%)	
LIC	2	78.34	77.5	68.34	74.73
DOU	2	57.14	62.86	71.43	63.81
GTL	2	42.86	66.7	88.4	65.99

^1^ LIC (licorice), DOU (doum), and GTL (green tea leaves).

^2^ People who tasted these samples were among 30–40 years, healthy, males and live at the Alexandria Province.

## 4 CONCLUSIONS

The current study demonstrated how the soaking procedure significantly aids the mycotoxin adsorption in cow and buffalo milk. The mycotoxin kind, the plant adsorbent agent, the applied concentration, and the milk constituents all impacted the method's effectiveness. At concentrations of 0.5% and 1%, GTLs completely adsorbed Zear particles. Contrarily, at a level of 1% in cow milk, LIC absorbed Dias particles; however, its efficiency was considerably diminished in the case of buffalo milk. Due to the mycotoxin particles' possible aggregation during the shacking process, the milk shacking method followed by filtering has only a modest impact in reducing mycotoxin particles. The incorporated plant fibers with the shacking process in contaminated milk augmented the effectiveness capacity to bind and adsorb the different particles of mycotoxins. In the cow milk case, GTL (1%) and shacking speed 200 rpm were adsorbed aflaM1, Zear and Dias particles. Still, the same plant fibers at level 2% and shacking speed 500 rpm were completely adsorbed CPA and Dias in milk‐tested samples. Whereas LIC (2%) combined with shaking speed 200 rpm were adsorbed CPA and Dias particles, at level 1% entirely adsorbed Zear particles. In the buffalo milk case, LIC and DOU exhibited the best efficacy ratios in removing mycotoxins than GTL under all the tested concentrations combined with shacking speed (200 or 500 rpm), where aflaM1 and Don were highly removed with GLT at levels 0.5% and 1%, respectively. Overall, the bio‐adsorption and biodegradation of mycotoxins using plant fibers have been proven to be a low‐cost and safe way to remove mycotoxins.

## CONFLICT OF INTEREST STATEMENT

The authors declare no conflict of interest in this paper.

## Data Availability

The data that support the findings of this study are available on request from the corresponding author. The data are not publicly available due to privacy restrictions.
